# Preparation and Physicochemical Characterization of Gelatin–Aldehyde Derivatives

**DOI:** 10.3390/molecules27207003

**Published:** 2022-10-18

**Authors:** Mahmoud Atya El-Meligy, Katarína Valachová, Ivo Juránek, Tamer M. Tamer, Ladislav Šoltés

**Affiliations:** 1 Centre of Experimental Medicine of SAS, Dúbravská cesta 9, 84104 Bratislava, Slovakia; 2 Polymer Research Group, Department of Chemistry, Faculty of Science, University of Tanta, Tanta 31527, Egypt; 3 Polymer Materials Research Department Advanced Technologies and New Materials Research Institute (ATNMRI), City of Scientific Research and Technological Applications (SRTA-City), New Borg El-Arab City P.O. Box 21934, Egypt

**Keywords:** FT-IR spectroscopy, gelatin functionalization, rotational viscometry, scanning electron microscopy, UV-Vis spectrometry, thermoanalytical methods

## Abstract

The present study aimed at preparing novel free-radical scavenging and water-soluble compounds derived from gelatin. Specifically, gelatin–syringaldehyde, gelatin–anisaldehyde, and gelatin–vanillin were synthesized and thoroughly studied for their physicochemical properties. In particular, the compounds were characterized by UV-Vis spectroscopy, Fourier-transform infrared spectroscopy, and scanning electron microscopy. Notably, as demonstrated by thermogravimetry and differential scanning calorimetry, all three derivatives exhibited higher thermal stability than gelatin itself. Free-radical scavenging activities of the examined compounds were explored by (i) a standard spectrophotometric ABTS assay and (ii) an assay of oxidative degradation of hyaluronic acid monitored by rotational viscometry. We found that gelatin and gelatin–syringaldehyde demonstrated the highest efficacy in scavenging ^•^OH radicals, whereas gelatin–anisaldehyde was the least effective. The efficacy of scavenging alkyloxy- and alkylperoxy-type free radicals via hydrogen-atom-transferring property was in the following order: gelatin > gelatin–vanillin > gelatin–syringaldehyde > gelatin–anisaldehyde. Electron-donor properties determined using the ABTS assay revealed the following order in one-electron reduction of ABTS^•+^: gelatin > gelatin–anisaldehyde > gelatin–vanillin > gelatin–syringaldehyde.

## 1. Introduction

**Figure 1:**
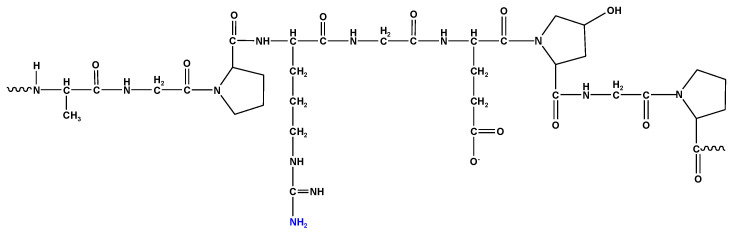
Chemical structure of gelatin.

Collagen is a water-insoluble protein presented in animals’ connective tissues such as tendons, cartilage, and bones [1]. Gelatin types A and B (Figure 1) are produced by the thermal denaturation of collagen under acidic or alkaline conditions, respectively [2,3]. The strength and stiffness of gelatin are determined by the bloom value [4].

Gelatin is a low-cost, commercially available material [5]. Compared to collagen, gelatin was found to possess low immunogenicity [3] and high solubility in aqueous environments [6]. It has film-forming, non-toxic, biodegradable, biocompatible, and hemostatic properties [7]. Currently, gelatin has been used in medicine as a wound-dressing material, drug carrier, and biomaterial for reconstructing blood vessels [8,9,10]. Gelatin forms a 3D porous structure when interacting with positively charged polymers, such as chitosan, and with ^•^OH groups in polyvinyl alcohol [11,12]. Furthermore, gelatin is rich in domains that readily interact with cell-surface receptors and other proteins of the extracellular matrix (ECM), such as fibronectin [2].

There are various ways to modify native gelatin, including modifications by phenolic aldehydes, among others, and those performed with p-anisaldehyde, syringaldehyde, or vanillin. The modification reaction is based on the immobilization of the reactant to the matrix of biopolymers by combining the phenolic aldehyde group with free –NH_2_ groups of gelatin. These modifications resulted in an improvement in the antibacterial properties of the given gelatin derivatives [3,9,13].

p-Anisaldehyde (4-methoxybenzaldehyde), one of the isomers of anisaldehyde, is an organic compound commonly found in synthetic and natural fragrances [14]. It is a major essential oil compound extracted from the seeds of Pimpinella anisum [15]; commercially, it is produced by the oxidation of methoxytoluene [16]. Due to its antibacterial and antifungal properties, p-anisaldehyde has many applications in medicine and pharmacology [16,17].

Syringaldehyde (3,5-dimethoxy-4-hydroxybenzaldehyde), belonging to the group of flavonoids, is a naturally occurring compound with various bioactive characteristics. Major natural sources of syringaldehyde are lignin and the cell wall of such plants as Manihot esculenta and Magnolia officinalis [2,18]. Syringaldehyde has a strong anti-inflammatory activity via inhibiting cyclooxygenase 2, as demonstrated, e.g., in a mouse macrophage cell line [19].

Vanillin, a natural phenolic compound extracted from the pods of Vanilla planifolia orchid, is one of the most extensively used flavoring agents in the food, beverage, and cosmetics industries [10]. Importantly, vanillin has been recognized as an essential bioactive compound possessing antioxidant, antimicrobial, antifungal, nephroprotective, antiviral, cardioprotective, and antitumor properties. It serves as a treating agent for a variety of diseases ranging from skin wounds to sickle cell anemia [20]. Furthermore, vanillin and its synthetic counterparts have been found to control gene expression, inhibit the excessive production of pro-inflammatory mediators and free radicals, and facilitate tissue regeneration [11,12,21].

Hyaluronan or hyaluronic acid (HA) is a prominent high-molar-mass linear glycosaminoglycan found in ECM, reaching a size of up to 8 MDa. The adult human body contains about 12–15 g of HA, most of which occurs in connective tissue, including skin, the vitreous body of the eye, synovial fluid of articular joints, intervertebral disks, embryonic mesenchymal tissues, and umbilical cord. Hyaluronan is involved in a number of processes, including skin wound healing, tissue repair and regeneration, organization of ECM, joint lubrication, regulation of cell adhesion and motility through receptors that interact with the cytoskeleton, angiogenesis via promoting cell proliferation, cell differentiation, and cell migration. HA also possesses immunomodulatory, anticancer, and antiproliferative properties [22].

Both gelatin and HA are appropriate candidates for pharmaceutical and medical applications, including tissue engineering and regenerative medicine. Their ability to mimic the architecture of ECM has been reported for respective hybrid hydrogels of gelatin and HA. Scaffolds consisting of both polymers were prepared and used for culturing a variety of cells, including vascular endothelial cells, myoblasts, osteoblasts, and stem cells [23].

The aim of the present study was: (i) to synthesize derivatives of gelatin by coupling it with naturally occurring aldehyde compounds, namely, p-anisaldehyde, vanillin, and syringaldehyde, and (ii) to investigate their properties in scavenging reactive oxygen species such as ^•^OH, alkyloxy- and, alkylperoxy-type radicals using the so-called hydrogen-atom-transferring (HAT) assay and free-electron donating assay via quenching the ABTS^•+^ probe.

## 2. Results and Discussion

**Figure 2:**
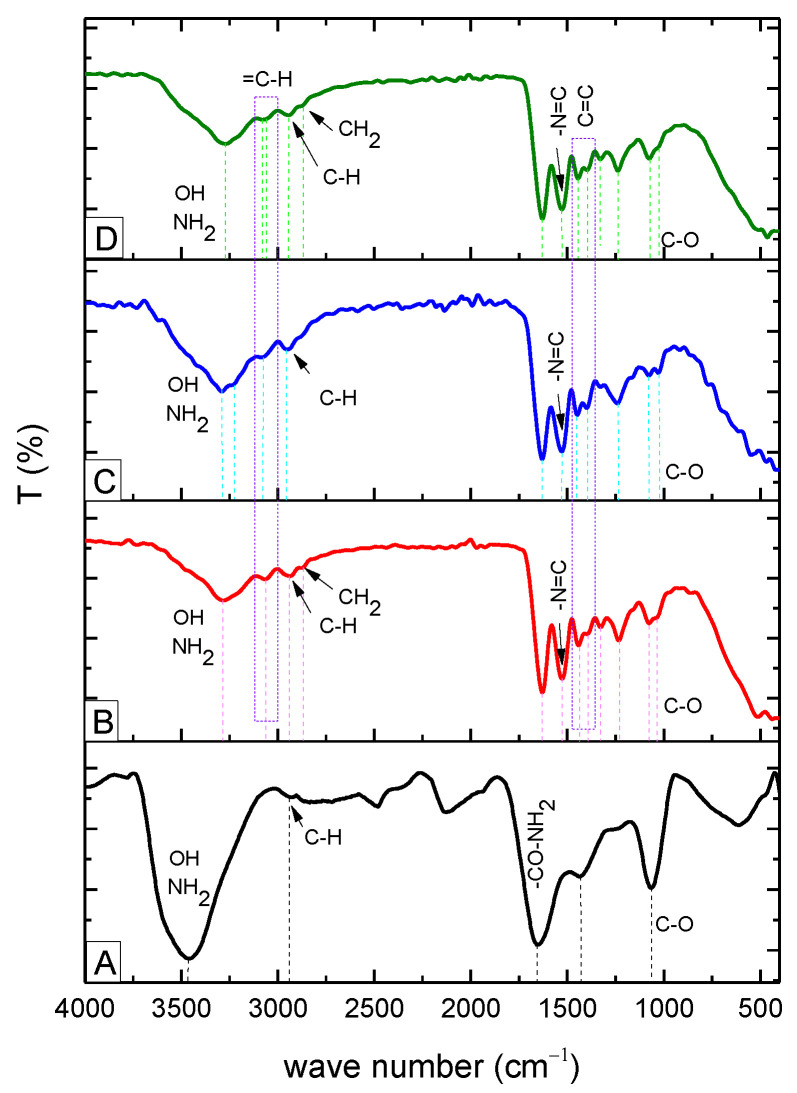
FT-IR of gelatin (**A**), gelatin–anisaldehyde (**B**), gelatin–syringaldehyde (**C**), and gelatin–vanillin (**D**).

The results of FT-IR depicted in Figure 2 showed that the absorption peaks observed at 3439 cm^−1^ for gelatin and 3282 cm^−1^ for the new gelatin derivatives correspond to the intermolecular hydrogen-bonded –OH stretching and –NH stretching in secondary amides (gelatin amide A) [24]. For pure gelatin, the peak obtained near 1635 cm^−1^ confirmed the presence of –C=O stretching in amides (amide I band). Further, distinct absorption bands obtained around 1430 cm^−1^ in the range of 1560–1335 cm^−1^ corresponded to the N–H bending in secondary amides (amide II bands) [25]. For the new gelatin derivatives, there was a significant peak generated as a result of the substitution of new functional groups. The peak near 1635 cm^−1^ was shifted to 1630 cm^−1,^ and a new peak for –C=N formation was generated due to the interaction of amino groups with aldehyde groups of vanillin (at 1527 cm^−1^), p-anisaldehyde (1526 cm^−1^), or syringaldehyde (1527 cm^−1^). In particular, characteristic aromatic bands C=C were observed in the range of 1400–1440 cm^−1^ in combination with the N–H bending in secondary amides (amide II bands). In addition, the stretching vibration of =C–H in the aromatic ring was observed at 3062 cm^−1^ for gelatin–anisaldehyde, at 3075 cm^−1^ for gelatin–syringaldehyde, and at 3080 and 3059 cm^−1^ for gelatin–vanillin. The bands in the range of 1240–670 cm^−1^ were attributed to the amide III region of gelatin [25,26]. The FT-IR results thus demonstrated the chemical reaction between gelatin and the aldehydes, and the respective peaks confirmed the formation of new imine linkages of Schiff bases in the range of 1630–1530 cm^−1^ for all derivatives. Furthermore, the peaks around 1440 cm^−1^ confirmed the formation of the gelatin Schiff base [27,28].

**Figure 3:**
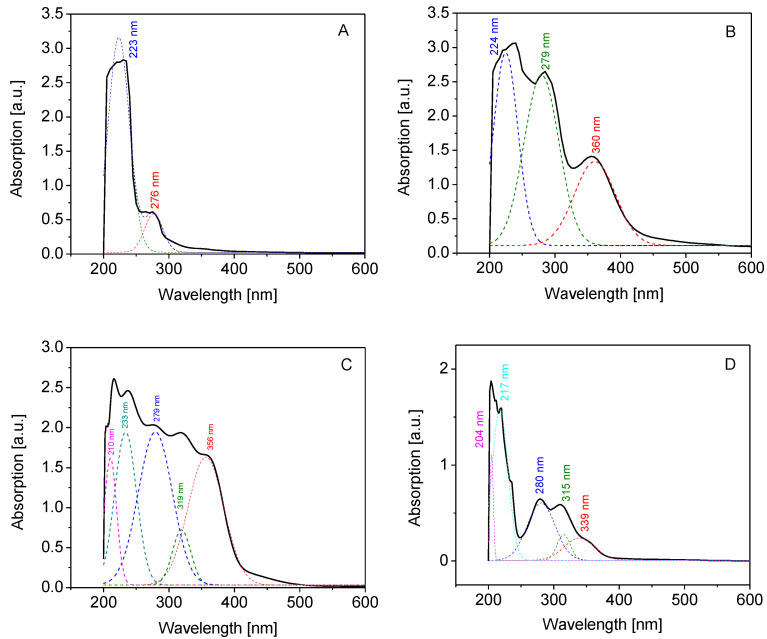
UV-Vis spectra of gelatin (**A**), gelatin–anisaldehyde (**B**), gelatin–syringaldehyde (**C**), and gelatin–vanillin (**D**).

The UV-visible spectra of gelatin (Figure 3A) and three selected representants of gelatin derivatives are depicted in Figure 3B–D. It was shown that bovine skin gelatin and modified gelatin gave a characteristic peak at 223 nm that reflected the percentages of specific amino acids in their content, namely, glycine (25.96%), proline (15.14%), arginine (11.30%), glutamic acid (8.17%), alanine (7.93%), phenylalanine (6.49%), tyrosine (6.25%), and other amino acids (18.75%) [29]. By substitution, free amine groups were coupled with conjugated phenyl groups that donated nitrogen with the stream of electrons through conjugated double bonds. As a result, the intensity of the peak increased, and its position shifted to a higher wavelength, as demonstrated by three gelatin derivatives, namely, gelatin–anisaldehyde (Figure 3B), gelatin–syringaldehyde (Figure 3C), and gelatin–vanillin (Figure 3D). Coupling gelatin amine groups with aromatic aldehydes immobilized a new nucleus along the gelatin backbone. The rise in the peak could be explained by the increased donor ability to stabilize the excited state. In addition, one new peak appeared at 360 nm for gelatin–anisaldehyde; two new peaks at 319 and 356 nm for gelatin–syringaldehyde; and three peaks at 280, 315, and 339 nm for gelatin–vanillin. This result was explained as the result of the generation of a new transition n–π*, forming a Schiff base bond –C=N between the amine of the gelatin and the carbonyl of the aromatic aldehyde [26,30]. 

**Figure 4:**
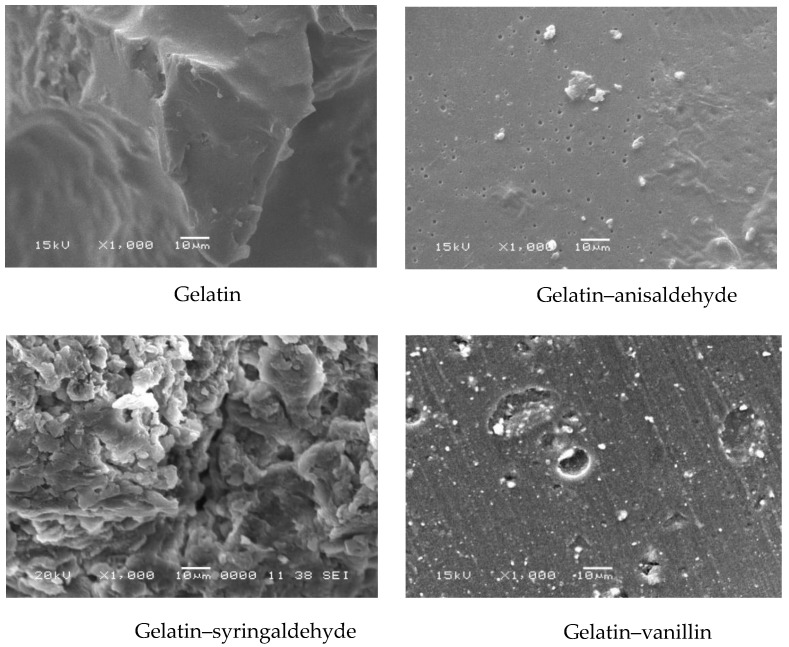
SEM of gelatin and gelatin derivatives.

Figure 4 shows the microstructure of gelatin, gelatin–anisaldehyde, and gelatin–syringaldehyde surfaces. There are visible porosity and cavities of pores on the surface created by coupling amine groups with the aromatic aldehyde. The smooth structure of gelatin may be attributed to the intermolecular bonds between its amino acid functional groups. The substitution of the macromolecules with aromatic Schiff bases allows them to re-esterify those bonds and build new intermolecular bonds. Microstructure alterations may be associated with the distortion of chain alignment and/or solvent evaporation during processing. Notably, the topography of biomaterials’ surfaces at the micro- and nano-scale influences the expression of the genes responsible for cell alignment, migration, differentiation, and proliferation [31,32,33]. It has been reported that when using various biomaterials as tissue scaffolds, the biomaterials’ porousness was an important factor in modulating tissue regeneration and wound healing, as demonstrated by the facilitation of dermal fibroblast adhesion and skin integration [34].

**Figure 5:**
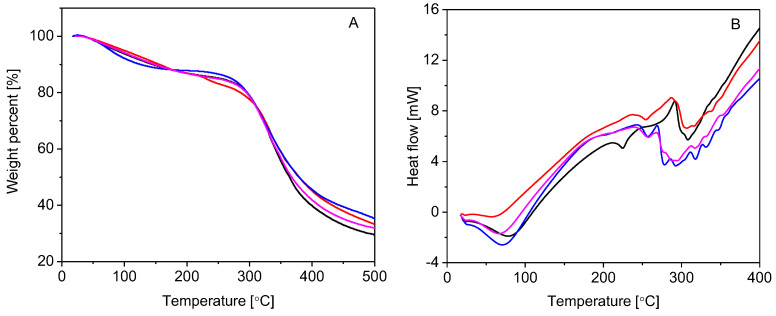
Thermal gravimetry (**A**) and differential scanning calorimetry (**B**) of gelatin (black), gelatin–anisaldehyde (red), gelatin–syringaldehyde (blue), and gelatin–vanillin (violet).

The thermal stability of gelatin and three selected gelatin derivatives, characterized in detail by SEM (cf. Figure 4), was specifically assessed by thermal gravimetry analysis (TGA), as depicted in Figure 5A. The parent compound and its two derivatives underwent three main stages of weight loss. The first stage of weight loss, matching the loss of free and bound water, appeared in the range of 40–50 °C up to approximately 150 °C. The moisture loss was 10.28% for gelatin, 9.50% for gelatin–anisaldehyde, 11.21% for gelatin–syringaldehyde, and 10.15% for gelatin–vanillin. In contrast to gelatin, a decrease in binding water by gelatin–anisaldehyde might be attributed to an increased hydrophobic character after coupling gelatin with anisaldehyde. On the other hand, compared to gelatin, a significant increase in the moisture content in gelatin–syringaldehyde was found. This increase may be explained by the pseudohydrophilic character of the derivative responsible for increasing the pore size of the sample, causing it to reduce moisture in the environment during the preparation process. The second stage of weight loss, associated with the loss of lower-molecular-weight proteins, was observed at the temperature range of 280–380 °C. Gelatin’s derivatives were observed to have increased thermal stability relative to gelatin. The third stage of weight loss (at a temperature > 400 °C) reflected the thermal decomposition of gelatin chains. 

Differential scanning calorimetry (DSC, Figure 5B) analysis of gelatin revealed an endothermic peak band around 100 °C, which was attributed to the evaporation of water molecules trapped by its hydrophilic groups (i.e., amine, carboxylic, and hydroxyl groups) [35,36]. A decrease in moisture content was observed in gelatin–anisaldehyde. The endothermic peak at a temperature of 216 °C is related to the thermal unfolding of the partial helical structure of gelatin. These bands were shifted and disappeared by replacement with cinnamyl groups. Gelatin decomposed at 230 °C—the point at which the exothermic peak was observed. A shift in the exothermic decomposition peak was attributed to the phenolic nucleus attached to gelatin and indicated the thermal stabilities of the formed Schiff bases.

**Figure 6:**
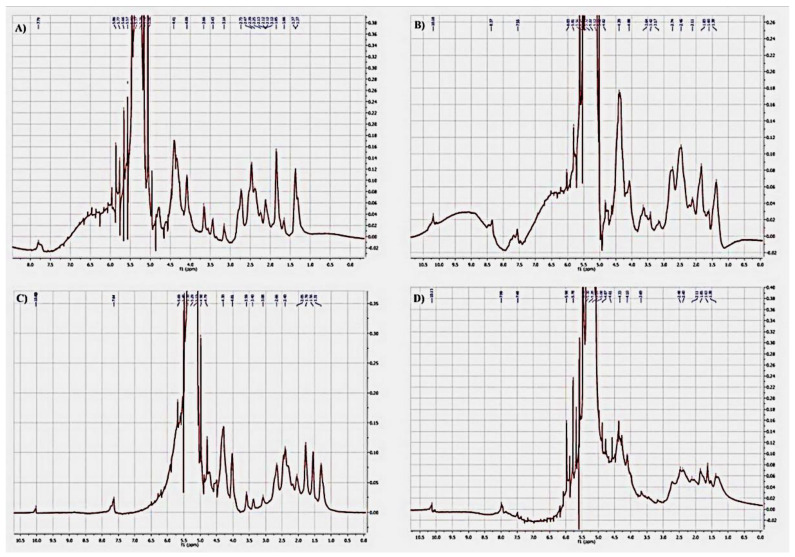
^1^H NMR analysis of gelatin (**A**), gelatin-anisaldehyde (**B**), gelatin-syringaldehyde (**C**) and gelatin-vanillin (**D**).

A ^1^H NMR analysis of gelatin, gelatin–anisaldehyde, gelatin–syringaldehyde, and gelatin–vanillin was performed Figure 6). Most of the proton signals could be assigned to the corresponding proton of the gelatin amino acids. In detail, the signal at δ 1.3–1.4 ppm was a β methylene group in (Ala); the signal at δ 1.5–1.6 ppm referred to γ methylene proton in (Arg); and multiple signals δ 1.8–2.6 ppm were a combination of a proton of β(CH_2_) Arg at δ 1.79 ppm, δCH_2_ (Arg) at δ 2.65 ppm, βCH_2_ Glut at δ 2.06 ppm, γCH_2_ (Glu) at δ 2.33 ppm, and γCH_2_ (Pro) at δ 2.2–2.3 ppm. Hydroxyl protons, αCH_2_ (Ala) and δCH (Pro), were at δ 3.5–3.7 ppm. The signal at δ 4.09 referred to αCH_2_ (Gly). The signal at δ 4.4 ppm referred to αCH_2_ (Pro) [37]. The coupling of gelatin and formation of aromatic Schiff base derivatives demonstrated a clear signal at δ7.5–8 ppm referring to aromatic protons and a signal δ 9.5–10 ppm referring to Schiff base proton –CH=N– [38,39].

**Figure 7:**
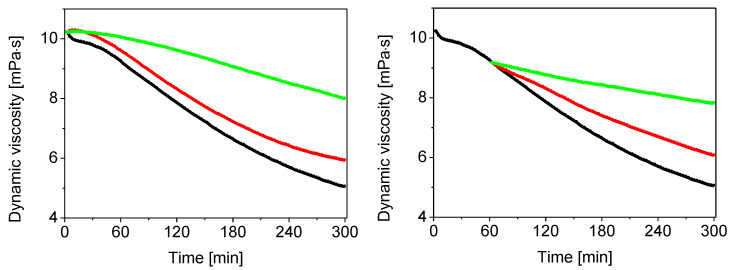
Time-dependent changes in dynamic viscosity of high-molar-mass HA solutions exposed to Cu(II) ions (1 µM) and ascorbate (100 µM) (black curve) and after the addition of gelatin (10 mg/mL) in volume 100 µL (red curve) and 1000 µL (green curve). Gelatin was added either before HA degradation began (left panel) or 1 h later (right panel).

Results in Figure 7, left panel (black curve), showed rapid ^•^OH radical-induced HA degradation. The decrease in dynamic viscosity of the HA solution was 5.2 mPa · s within 5 h. When the gelatin solution was added in the volume of 100 µL, minimal prevention of HA oxidative degradation was seen (red curve). The addition of 1000 µL of the gelatin solution (green curve) resulted in a more significant inhibition of HA degradation. The dynamic viscosity of the HA solution dropped by only 2.22 mPa · s. Similar results are shown in Figure 7, right panel, where the propagation of the HA degradation was mediated by alkyloxy- and/or alkylperoxy-type radicals. The addition of gelatin in volumes of 100 (red curve) and 1000 µL (green curve) dose-dependently reduced the rate of HA degradation, whereas the decreases in the dynamic viscosity of the HA solution were 2.7 and 1.35 mPa · s, respectively.

Table 1 summarizes the percentage of scavenging potency towards the reactive oxygen species of gelatin and its three derivatives. Concerning the elimination of ^•^OH radicals, gelatin and gelatin–syringaldehyde were shown to be the most potent: they scavenged 57% of these radicals. A slightly weaker effect was shown for the derivatives gelatin–vanillin (52%) and gelatin–anisaldehyde (45%). As for the scavenging of alkyloxy- and alkylperoxy-type radicals, the most potent of these was gelatin, followed by gelatin–vanillin, gelatin–syringaldehyde, and gelatin–anisaldehyde. These results indicate that gelatin and its derivatives exerted their H-atom-transferring properties.

**Figure 8:**
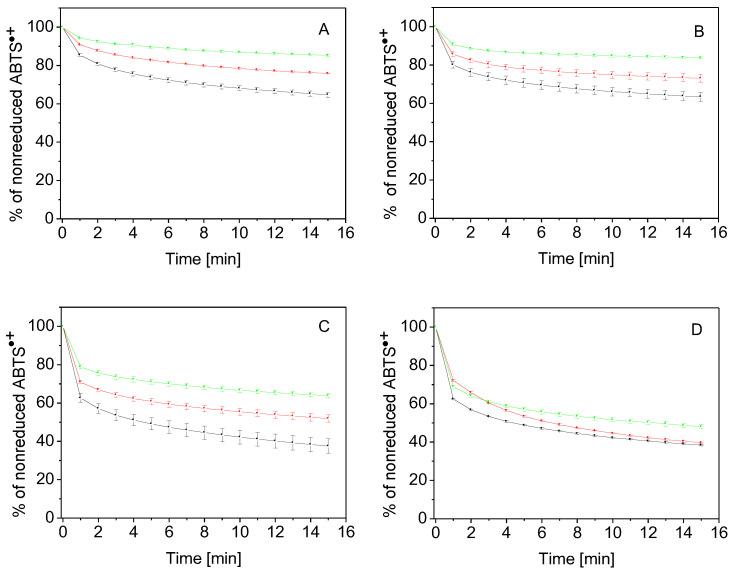
Time-dependent changes in the kinetics of the reduction of ABTS^•+^ by gelatin (**A**), gelatin–anisaldehyde (**B**), gelatin–syringaldehyde (**C**), and gelatin–vanillin (**D**), all added at concentrations of 5 (green), 10 (red), and 20 mg/mL (black). The measurements were performed in triplicate by spectrophotometry at 734 nm within 15 min. The time dependencies of the percentage of nonreduced ABTS^•+^ pass through the means of the measurements; as a result, the SEMs are indicated by the standard method.

As depicted in Figure 8, gelatin (panel A) and gelatin–anisaldehyde (panel B) showed poor electron-donor properties. The nonreduced ABTS^•+^ percentages were 64.7% and 63.3%, respectively, when gelatin and gelatin–anisaldehyde were added at their highest concentration, i.e., 20 mg/mL. In comparison, gelatin–syringaldehyde (panel C) revealed a higher potency for scavenging ABTS^•+^. The amounts of unscavenged ABTS^•+^ were 68%, 50%, and 40% when adding this compound at concentrations of 5, 10, and 20 mg/mL, respectively. Finally, gelatin–vanillin was found to be the most effective derivative for scavenging ABTS^•+^ (panel D); the amounts of unscavenged ABTS^•+^ were the lowest among the examined compounds. Thus, gelatin–vanillin was revealed to be the most effective electron donor compound of all the tested compounds in the study.

The novelty of the present study is the design, preparation, and essential physicochemical characterization of the novel gelatin-based aldehydes. Along with spectrophotometric methods (FT-IR, UV/Vis, ^1^H NMR, thermoanalytical methods), thermogravimetry, differential scanning calorimetry, and the microscopic method (SEM), an original rotational viscometric method has been employed to examine the free-radical properties of all four compounds. The latter method has been applied to compare free-radical inhibition properties between native gelatin and novel gelatin–aldehyde derivatives.

The results of the present study demonstrated that gelatin and its derivatives were effective at given concentrations in inhibiting reactive-oxygen-species-mediated HA degradation. Few papers report the efficacy of gelatin for scavenging ^•^OH radicals. He et al. (2002) [40] and Xiao et al. (2007) [41], using electron paramagnetic resonance analysis, showed that type I collagen in the concentration range of 10–150 µg/mL dose-dependently inhibited ^•^OH generation in Fe^2+^-mediated Fenton reaction. In contrast, gelatin in the same concentration range hardly inhibited formed ^•^OH radicals. He et al. (2002) [40] showed that type I collagen at a concentration of 150 µg/mL inhibited ^•^OH-induced apoptosis of HeLa cells more effectively than gelatin.

## 3. Materials and Methods

### 3.1. Materials

High-molar-mass hyaluronan (M_w_ = 1.69 MDa, M_w_/M_n_ = 1.63) was purchased from Lifecore Biomedical Inc., Chaska, MN, USA. Gelatin powder (type B; bovine skin; 300 bloom) was purchased from Fluka, Buchs, Switzerland. Syringaldehyde (98%), vanillin (98%), and p-anisaldehyde (98%) were supplied from Chemical Engineering Co., Beijing, China. CuCl_2_·H_2_O p.a., NaCl p.a., ethanol p.a. (96%), and acetone p.a. were purchased from Slavus Ltd., Košice, Slovakia. Ascorbic acid was from Merck KGaA, Germany. 2,2′-Azinobis-(3-ethylbenzothiazoline)-6-sulfonic acid (ABTS) diammonium salt was obtained from Sigma-Aldrich Chemie GmbH, Steinheim, Germany. Deionized high-purity-grade water, with a conductivity of ≤0.055 µS/cm, was made using the TKA water purification system (Water Purification Systems GmbH, Berlin, Germany).

### 3.2. Methods

#### 3.2.1. Synthesis of Gelatin Derivatives Anisaldehyde–, Vanillin– and Syringaldehyde–Gelatin

**Figure 9:**
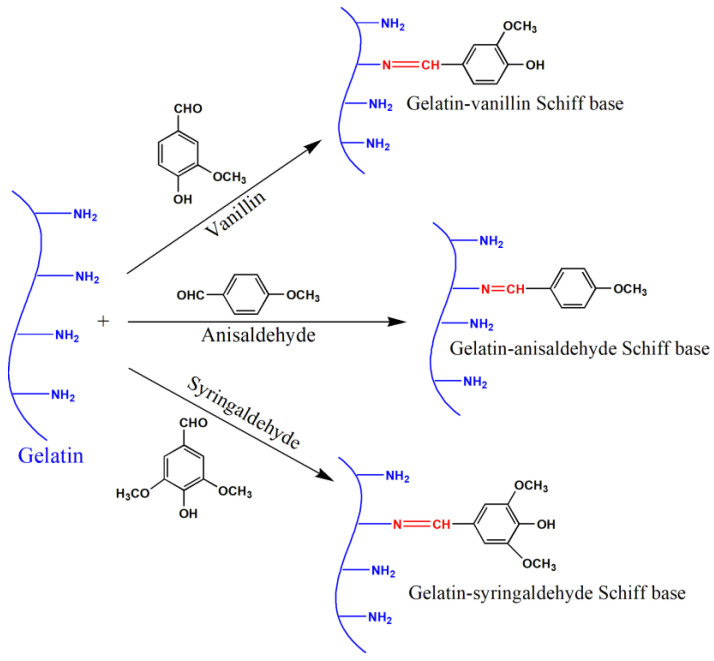
The preparation of the gelatin–vanillin, gelatin–anisaldehyde, and gelatin–syringaldehyde derivatives.

Bovine type B gelatin was modified by coupling its free amine groups with p-anisaldehyde, vanillin, or syringaldehyde as follows: 

One gram of gelatin was dissolved in 20 mL distilled water and stirred at 50 °C until forming a colorless homogenous solution. Then, 10 mL of ethanol was added dropwise to the solution during mixing (to avoid the quick gelation of the polymer during the reaction). Further, 0.3 g of the given aldehyde was dissolved in 10 mL of ethanol at ambient temperature, followed by dropwise addition to gelatin solution under stirring. The mixture was heated to 70 °C and stirred for 6 h, and the solution converted from colorless or faintly yellow to yellow due to the formation of a Schiff base. Finally, acetone was added to form a precipitate that was subsequently isolated from the reaction mixture by filtration. The final product was washed several times with ethanol to remove the excess aldehyde reagents and was then dried at 60 °C in an oven. The product yields ranged from 94% to 99%. Figure 9 represents the synthesis of gelatin–vanillin, gelatin–anisaldehyde, and gelatin–syringaldehyde Schiff base derivatives.

#### 3.2.2. Fourier-Transform Infrared Spectrophotometry (FT-IR)

A Fourier-transform infrared spectrophotometer (Shimadzu FTIR–8400 S, Shimadzu Scientific Instruments Inc., Kyoto, Japan) was used to analyze gelatin and its novel derivatives. All samples were freeze-dried using liquid nitrogen, crushed to a fine powder (KBr: sample = 140:2 mg/mg), and pressed into a transparent disk with a diameter of 13 mm by applying a 105 N force. The FT-IR spectra were obtained by recording 64 scans from 4000 to 400 cm^−1^ with a resolution of 2 cm^−1^ in absorbance mode.

#### 3.2.3. UV-Vis Spectroscopic Analysis 

The UV-Vis spectra of the gelatin and Schiff base derivatives were determined using a spectrophotometer (Model Ultrospec 2000, Pharmacia Biotech, Uppsala, Sweden). The samples of gelatin and gelatin derivatives (0.05 g) were dissolved in 10 mL of distilled water under heating. Absorbances of the solutions in a quartz cell were scanned from 200 to 600 nm.

#### 3.2.4. Scanning Electron Microscopy Analysis 

Scanning electron microscopy (SEM) was applied to examine gelatin, gelatin–anisaldehyde, and gelatin–syringaldehyde after coating them with a thin layer of gold under vacuum. Morphological changes of the sample’s surface were followed using a secondary electron detector of SEM (Joel Jsm 6360LA, Jeol Ltd, Tokyo, Japan.

#### 3.2.5. Thermal Gravimetric Analysis 

Thermal stability of gelatin and its derivatives (~6 mg) was carried out using a thermogravimetric analyzer device (Shimadzu TGA –50/50H, Shimadzu, Tokyo, Japan) at a temperature range of 25–500 °C with a heating rate of 10 °C/min under nitrogen flow (20 mL/min).

#### 3.2.6. Differential Scanning Calorimetry

Differential scanning calorimetric analysis of gelatin and its derivatives (~5 mg in a sealed aluminum pan) was carried out using a differential scanning calorimeter device (Shimadzu DSC–60A, Shimadzu, Tokyo, Japan) at a temperature range of 25–400 °C with a heating rate of 10 °C/min under nitrogen flow (30 mL/min).

#### 3.2.7. ^1^H NMR Analysis

The ^1^H NMR spectra of gelatin and novel gelatin derivative molecules were obtained using 500 MH Jeol (Jeol Ltd, Tokyo, Japan). ^1^H NMR spectra were also obtained separately in 0.5 wt. % D_2_O to characterize the structure of the molecules.

#### 3.2.8. Effects on Free-Radical-Mediated Degradation of Hyaluronan

Hyaluronan (HA) was dissolved overnight in 0.15 M aqueous NaCl as follows: First, 4.0 mL of solvent was added to HA (16 mg). Then, after 6 h, an additional 3.9, 3.8, or 2.9 mL of 0.15 M NaCl was added. Stock solutions of ascorbic acid (16 mM) and cupric chloride (160 µM) were prepared in 0.15 M NaCl. To prepare stock solutions of gelatin and its derivatives, the compounds (0.1 g) were dissolved in 10 mL of deionized water under heating at 50 °C.

HA degradation was induced by the oxidative system consisting of CuCl_2_ (1 µM) and ascorbic acid (100 µM). The procedure was as follows: CuCl_2_ stock solution (50 µL) was added to the HA solution (7.90 mL) and stirred for 30 s. After 7.5 min without stirring the reaction mixture, the stock solution of ascorbic acid (50 µL) was added to the HA solution, stirred for 30 s, and the mixture was immediately transferred into the viscometer Teflon^®^ vessel.

Procedures to explore gelatin and its derivatives as inhibitors of HA degradation were as follows:

(a) In the first set of experiments, the stock solution of CuCl_2_ (50 µL) was added to the HA solution (7.8 or 6.9 mL) and stirred for 30 s. After 7.5 min without stirring, 100 or 1000 µL of gelatin or its derivatives was added and stirred for 30 s. Finally, the stock solution of ascorbic acid (50 µL) was added, stirred for 30 s, and the reaction mixture was immediately transferred into the viscometer Teflon^®^ vessel. Under these conditions, the compounds were examined for their ability to inhibit the initiation phase of HA degradation.
(b) In the second set of experiments, a similar procedure as described in (a) was applied. However, after 7.5 min, 50 µL of stock solution of ascorbic acid was added, followed by stirring the HA mixture for 1 h. Then, 50 µL of the stock solution of gelatin or its derivatives was added and stirred for 30 s. The HA solution was immediately transferred into the viscometer Teflon^®^ vessel. Under these conditions, the compounds were tested for their ability to inhibit the propagation phase of HA degradation.

The changes in the dynamic viscosity value were monitored for 5 h. The parameters of the measurements were 180 rpm, a shear rate of 237 s^−1^, and a temperature of 25 °C. The data were recorded in 3 min intervals using a rotational viscometer (Brookfield Engineering Labs, Inc., Middleboro, MA, USA).

#### 3.2.9. ABTS Assay

ABTS^●+^ probe was prepared at room temperature 24 h before the measurements as follows: ABTS aqueous stock solution (7 mM) was mixed with K_2_S_2_O_8_ aqueous solution (2.45 mM) in the equivolume ratio. On the next day, 1 mL of the generated ABTS^●+^ solution was diluted with deionized high-purity grade water to the volume of 60 mL [42,43].

Gelatin and its derivatives were dissolved in water yielding the stock concentrations of 20 mg/mL. These solutions were diluted with water to reduced concentrations of 10 and 5 mg/mL. For the measurements, 2 mL of the ABTS^●+^ solution was mixed with 50 µL of the stock and working solutions of gelatin and its derivatives (20, 10, and 5 mg/mL). The light absorbance was recorded in triplicate within 15 min at the wavelength of 734 nm using UV-Vis 1800 spectrophotometer (Shimadzu, Tokyo, Japan).

## Figures and Tables

**Table 1:** Reactive oxygen species scavenging activity of gelatin and its derivatives when added into the reaction vessel at volume of 1000 μL.

Sample	^•^OH Radical Scavenging Activity (%)	Alkyloxy- and/or Alkylperoxy-Type Radical-Scavenging Activity (%)
Gelatin	57	67
Gelatin–anisaldehyde	45	45
Gelatin–syringaldehyde	57	50
Gelatin–vanillin	52	55

## Data Availability

Not applicable.

## Sample Availability

Samples of the compounds were available from M.A. El-Meligy.
